# Pulmonary Valve Replacement: Update on Timing and Ventricular Remodelling

**DOI:** 10.3390/jcm15031295

**Published:** 2026-02-06

**Authors:** Almudena Ortiz-Garrido, Monika Różewicz Juraszek, Dominik Daniel Gabbert, Jill Jussli-Melchers, Inga Voges

**Affiliations:** 1 Section of Paediatric Cardiology, Hospital Materno Infantil, Regional Universitario de Málaga, 29011 Malaga, Spain; 2 Faculty of Medicine, University of Málaga, 29010 Malaga, Spain; 3 Department of Congenital Heart Disease and Paediatric Cardiology, University Hospital Schleswig-Holstein, Campus Kiel, 24105 Kiel, Germany; 4 German Centre for Cardiovascular Research (DZHK), Partner Site Hamburg/Kiel/Lübeck, 24105 Kiel, Germany; 5 Department of Cardiac Surgery, Universitatsklinikum Schleswig-Holstein, 24105 Kiel, Germany; markajill.jussli-melchers@uksh.de

**Keywords:** pulmonary valve replacement, tetralogy of Fallot, pulmonary regurgitation, right ventricular remodelling, cardiovascular magnetic resonance, timing of intervention, arrhythmia, fibrosis

## Abstract

Chronic pulmonary regurgitation (PR) after the repair of tetralogy of Fallot (TOF) and other right ventricular outflow tract (RVOT) interventions leads to progressive right ventricular (RV) dilatation, altered ventricular–ventricular interaction, and an increased risk of arrhythmia and heart failure. Pulmonary valve replacement (PVR), whether surgical or transcatheter, effectively eliminates or reduces PR and is associated with short- and mid-term improvement in RV size, symptoms, and electrocardiographic markers. However, the optimal timing of intervention remains unresolved: operating late can result in irreversible myocardial damage and arrhythmogenic substrates, whereas operating early can lead to repeated reinterventions, the impact of which on hard outcomes is uncertain. This review summarizes contemporary evidence on ventricular remodelling after PVR, focusing on cardiovascular magnetic resonance (CMR) and echocardiographic markers, and critically appraises proposed criteria for timing PVR. Classic CMR-derived thresholds (RV end-diastolic volume index [RVEDVi] 150–170 mL/m^2^, RV end-systolic volume index [RVESVi] 80–90 mL/m^2^) and QRS duration cut-offs are discussed alongside emerging markers of risk, including the RV mass-to-volume ratio, diffuse myocardial fibrosis (extracellular volume fraction), strain imaging, and diastolic dysfunction. Meta-analyses show consistent reverse remodelling and symptomatic benefit after PVR, but no conclusive survival benefit has been demonstrated, and data on arrhythmic outcomes remain conflicting. Key gaps include (i) the lack of prospective randomized or carefully matched comparative studies of “early” versus “deferred” PVR; (ii) limited understanding of how myocardial fibrosis, RV hypertrophy, and diastolic dysfunction interact with volume load and timing to influence long-term outcomes; (iii) under-representation of adult and older adult TOF cohorts; and (iv) insufficient integration of multiparametric risk scores and machine-learning approaches into clinical decision-making. Future research should prioritize multicentre longitudinal cohorts with standardized imaging, electrophysiological and clinical endpoints, incorporate advanced imaging techniques (e.g., strain, 3D late gadolinium enhancement, and T1 mapping), and explore precision-medicine strategies to individualize PVR timing.

## 1. Introduction

Surgical repair of tetralogy of Fallot (TOF) and other right ventricular outflow tract (RVOT) lesions has transformed survival rates for this population. Most patients now reach adulthood in good health and enjoy an excellent quality of life. However, the relief of RVOT obstruction often comes at the cost of pulmonary valve incompetence, particularly in those who underwent surgical repair with a transannular patch. Chronic severe pulmonary regurgitation (PR) leads to progressive right ventricular (RV) volume overload, dilatation, altered geometry, and, eventually, systolic and diastolic dysfunction [1,2]. These changes are associated with reduced exercise capacity, heart failure symptoms, and an increased risk of ventricular arrhythmias and sudden cardiac death [3,4].

PVR most commonly occurs as a late consequence of surgical repair for TOF and other congenital heart diseases (CHDs) involving reconstruction of the RVOT. In these conditions, the relief of RVOT obstruction during childhood is often achieved using a transannular patch or an RV-to-pulmonary artery conduit. This inevitably results in chronic PR and progressive RV volume overload over time [1,3,4]. Given the improving long-term survival of patients with repaired CHD, the number of adolescents and adults living with chronic pulmonary valve dysfunction is steadily increasing worldwide. This makes PVR one of the most common reinterventions in the growing adult CHD population [5,6,7]. This growing global burden highlights the importance of optimizing the timing of PVR to balance the risk of irreversible ventricular remodelling with the cumulative burden of repeated interventions over a lifetime.

PVR is the mainstay of treatment for significant PR in this population. It can be performed surgically (with a homograft, xenograft, or other prosthesis) or percutaneously using transcatheter valves in suitable anatomies. Multiple observational studies and meta-analyses have shown that PVR reduces RV volumes, improves the NYHA class, and shortens QRS duration [5,6,7,8]. Yet, despite several decades of experience, there is no consensus on the optimal timing of PVR in asymptomatic or mildly symptomatic patients [9,10,11].

Traditional approaches have emphasized volumetric thresholds derived from cardiovascular magnetic resonance (CMR), parameters from electrocardiograms (ECG) such as QRS duration, symptom burden, and pragmatic considerations (e.g., availability of transcatheter options, need for concomitant surgical procedures) [1,2,7]. However, emerging data suggest that irreversible myocardial damage, including diffuse myocardial fibrosis and adverse changes in RV and left ventricular (LV) mechanics, may precede conventional thresholds based purely on ventricular volumes [12,13]. Conversely, recent work indicates that many patients with apparently “severe” RV dilatation remain clinically stable with preserved function for long periods, challenging a purely volume-driven “early PVR” paradigm [14].

This review aims to:

- Summarize the pathophysiological basis of RV remodelling in chronic PR.
- Review the evidence on ventricular remodelling following PVR (surgical and transcatheter).
- Critically evaluate proposed criteria and thresholds for timing PVR.
- Identify key gaps in knowledge and propose priorities for future research.

This review is structured to progress from the pathophysiology of chronic PR and ventricular remodelling, through imaging-based markers of myocardial health, to their integration into contemporary clinical decision-making regarding the timing of PVR.

## 2. Pathophysiology of Chronic PR: Ventricular Remodelling

### 2.1. Volume Overload and RV Adaptation

Following TOF repair, the pulmonary valve is sometimes absent or frequently severely incompetent. Chronic PR creates a pure volume overload state: during diastole, blood flows back from the pulmonary arteries into the RV, increasing the end-diastolic volume. The RV responds with eccentric hypertrophy and dilatation to maintain stroke volume and cardiac output [2].

Initially, this adaptation is beneficial: RV ejection fraction (RVEF) may be preserved or even supranormal, and patients often remain asymptomatic. However, experimental and, most importantly, clinical evidence suggests that prolonged severe PR eventually exceeds the adaptive capacity of the RV myocardium [2,13]. At this stage, myocyte slippage, extracellular matrix remodelling, and fibrosis lead to reduced contractile reserve, impaired ventricular–arterial coupling, and progressive RV dysfunction.

### 2.2. Ventricular–Ventricular Interaction and LV Impact

The RV and LV share a common interventricular septum and are constrained by the pericardium. Chronic RV dilatation leads to septal flattening and paradoxical motion, impairing LV filling and systolic performance [3,15]. CMR and echocardiographic studies have documented reduced LV longitudinal and circumferential strain in repaired TOF patients with marked RV dilatation, even when LVEF is preserved [3,16].

LV dysfunction is prognostically important. In a large multicentre cohort (INDICATOR), LV systolic dysfunction defined by reduced LVEF (LVEF z-score < −2) and measured by CMR volumetric analysis was an independent predictor of death and sustained VT, alongside RV hypertrophy and atrial arrhythmias [17]. This underscores that timing PVR purely to normalize RV volumes, without considering LV mechanics, may be insufficient.

### 2.3. QRS Duration, Scar, and Arrhythmogenic Substrate

The typical surgical repair leaves scars in the right ventricular outflow tract (RVOT) and the interventricular septum. These, together with RV dilatation, create a substrate for macro-reentrant ventricular tachycardia. Early work linked a QRS duration ≥ 180 ms to an increased risk of sudden death and sustained ventricular tachycardia (VT) [4]. However, later studies, including the INDICATOR study, showed that the QRS duration alone is a relatively weak predictor compared with combined models incorporating the RV mass-to-volume ratio and LV function [17], highlighting the importance of an integrated evaluation.

CMR late gadolinium enhancement (LGE) imaging is a technique that is able to detect focal areas of myocardial fibrosis within the RV and LV myocardium including the surgical sites as the RVOT and the interventricular septum. Three-dimensional (3D) LGE burden has been shown to correlate with inducible VT in TOF patients [18]. Diffuse fibrosis and the extracellular volume (ECV) fraction, as assessed by CMR T1 mapping, appear to provide additional independent prognostic information for adult patients, with a higher ECV being associated with arrhythmias and adverse events [13,19]. The interplay between volume load, conduction delay, and myocardial fibrosis is complex and remains incompletely characterized.

From a clinical perspective, these electrical and tissue markers are relevant to chronic PR because they are associated with progressive RV dilatation and remodelling. In turn, this is linked to QRS prolongation and may facilitate reentrant VT in the presence of surgical scarring [4,17,18]. However, the QRS duration and documented arrhythmias are not used as isolated triggers for PVR; rather, they influence timing decisions as part of an integrated assessment, particularly when PR is severe and accompanied by progressive QRS widening, atrial or ventricular arrhythmias, or evidence of an adverse myocardial substrate, such as extensive scarring or increased extracellular volume [13,17,18].

## 3. Clinical Imaging Assessment of Ventricular Remodelling

Whereas Section 2 focuses on the pathophysiological mechanisms underlying chronic PR and ventricular remodelling, this section summarizes the key imaging techniques and quantitative parameters used in routine clinical practice to assess these processes and guide decision-making.

### 3.1. Echocardiography and Strain

Echocardiography remains the first-line modality, especially for serial follow-up. Conventional indices include RV fractional area change, tricuspid annular plane systolic excursion (TAPSE), tissue Doppler S′, and estimates of RV systolic pressure [19,20]. However, these are angle-dependent and less accurate because of the complex RV geometry compared to the LV.

On the other hand, speckle-tracking echocardiography enables the quantification of longitudinal strain and the strain rate for both ventricles. Abnormal LV and RV longitudinal strain, even with preserved EF, has been associated with exercise intolerance and arrhythmic events in repaired TOF [3,16,21]. Early data suggest that impaired RV strain may precede overt volumetric deterioration and could be a more sensitive trigger for intensifying follow-up or considering PVR, but robust thresholds are not yet validated [21].

### 3.2. CMR

CMR is the reference standard for the measurement of RV volumes and function as well as PR quantification in repaired TOF [1,2].

Key CMR parameters normally include:

- RVEDVi and RVESVi (mL/m^2^);
- RVEF and LVEF (%);
- PR fraction (%) by phase-contrast imaging;
- RVOT aneurysm/akinesia and conduit/patch morphology;
- Myocardial structural changes: focal (LGE) and diffuse (T1 mapping/ECV).

Geva’s comprehensive review highlighted the central role of CMR in late TOF follow-up and PVR decision support, emphasizing that RV volumetric and functional data cannot be reliably obtained by echocardiography alone in many patients [2].

#### CMR Tissue Characterization

CMR LGE imaging identifies focal areas of scar tissue, which are strongly associated with adverse outcomes and inducible VT [17]. Diffuse fibrosis can be quantified using T1 mapping and ECV; increased LV or RV ECV has been linked to a worse functional status and arrhythmias in repaired TOF [13,19]. These techniques are promising tools to refine risk stratification and may help distinguish patients who can “tolerate” substantial RV dilatation from those in whom microscopic myocardial damage is progressing.

However, technical variability and the limited availability of longitudinal data currently prevent the routine incorporation of this technique into guidelines, particularly for younger patients.

Furthermore, the assessment of diffuse myocardial fibrosis in the RV using T1 mapping remains technically challenging. The thin RV free wall, complex geometry, and pronounced trabeculation increase susceptibility to partial volume effects and blood pool contamination, which can reduce the accuracy and reproducibility of measurements [22,23]. In addition, heart rate variability, respiratory motion, and differences in acquisition sequences and post-processing algorithms between vendors further limit the standardization of RV T1 and ECV measurements, particularly in multicentre studies [13,22].

## 4. Ventricular Remodelling After PVR

### 4.1. Prognostic Impact of RV Dilatation

Prospective and retrospective CMR series consistently show a rapid reduction in RV volumes after PVR. In a landmark pediatric study, Buechel et al. demonstrated that early surgical PVR (mean age ~14 years) led to significant reductions in RVEDVi and RVESVi (on the order of 30–40%) within six months, with modest improvement in RVEF [21]. Adult cohorts confirm a similar but less complete remodelling response [16].

Similarly, Hallbergson et al. reported on 101 repaired TOF patients followed up to 10 years after PVR [22]. Within the first post-operative year, PR fraction fell from ~49% to ~3%, RVEDVi decreased by ~39%, and RVESVi by ~33%. RVEF, interestingly, declined slightly in the early period, likely reflecting the shift from volume-loaded hyperdynamic function to more normal hemodynamics. These improvements in volume were largely stable for the first 5–6 years.

A meta-analysis of 3118 patients from 48 studies confirmed that PVR leads to significant improvement in RV volumes, RVEF, LVEF, QRS duration, and NYHA class, with low early mortality [5]. Similar findings have been reported in more recent meta-analyses [8].

### 4.2. Prognostic Significance of RV Dysfunction

Although RV volumes almost always decrease after PVR, normalization is not guaranteed. Oosterhof et al., in a CMR-based study of 71 adults, found that patients with pre-operative RVEDVi < 160 mL/m^2^ or RVESVi < 82 mL/m^2^ were more likely to normalize RV volumes after surgery [23]. These thresholds have been widely cited and incorporated into practice as targets for “timely” PVR. Multicentre data further support the prognostic relevance of these volumetric thresholds [24,25].

However, subsequent work has modulated this view. Hallbergson et al. showed that, beyond seven years after PVR, RV volumes began to increase again, approaching ~80–100% of pre-PVR values, accompanied by recurrent PR and elevated RV pressures [22]. This underscores that PVR is palliative: prosthetic valves eventually degenerate, RV loading conditions can deteriorate, and remodelling benefits may wane over time.

More recently, observational cohorts and registries suggest that pre-operative volumetric cut-offs alone do not reliably predict survival or arrhythmia risk and that some patients with apparently “excessive” RV dilatation experience meaningful remodelling and good clinical outcomes, whereas others with smaller volumes fare poorly due to adverse myocardial substrate [18,24,26].

### 4.3. LV Involvement and Clinical Outcomes

Surgical PVR has the longest track record, with decades of data showing acceptable peri-operative mortality (often <1–2%) and good mid-term durability [5,24,27]. Surgical approaches allow concomitant RVOT remodelling, tricuspid valve repair, relief of branch pulmonary artery stenoses, or the closure of residual shunts [7]. Large registry data demonstrate comparable early RV remodelling after TPVR [28].

Transcatheter PVR (TPVR), using valves such as Melody™ or Sapien™ devices and newer dedicated self-expanding valves for native RVOTs, has become an important alternative in appropriately sized RVOTs and conduits. TPVR avoids sternotomy and cardiopulmonary bypass, with high procedural success and low early morbidity [7,28]. CMR and echocardiography demonstrate a similar magnitude of early RV reverse remodelling with TPVR and surgical PVR when PR is abolished, though long-term comparative data are limited and influenced by differences in patient selection [6,26].

In both approaches, the degree of reverse remodelling appears to depend more on pre-operative RV geometry, associated lesions (e.g., RVOT aneurysm and tricuspid regurgitation), and myocardial integrity than on the choice of valve implantation technique [5,6,7,21].

### 4.4. Arrhythmic Risk and Electrical Remodelling

Meta-analyses and individual studies have highlighted that PVR not only benefits the RV but also improves LV function, particularly in those with pre-existing LV impairment [6,13,29]. Echocardiographic and CMR-derived strain data show the partial restoration of LV mechanics and better septal motion after PVR, although normalization is rare in patients with longstanding dysfunction [16,26].

These findings support the concept that PVR can interrupt but not fully reverse the adverse spiral of ventricular–ventricular interaction. Once diffuse myocardial fibrosis and maladaptive remodelling are advanced, the scope for recovery becomes limited, even if volume load is eliminated [13,19].

Tables S1–S3 summarize key studies and meta-analyses evaluating ventricular remodelling after surgical and transcatheter pulmonary valve replacement, including design, cohort characteristics, and principal remodelling findings.

## 5. Timing of PVR: Evidence, Controversies, and Guideline Thresholds

### 5.1. Traditional Volume- and Symptom-Based Thresholds

PVR is generally recommended in patients with repaired TOF and severe PR under the following circumstances [8,11]:

- Symptomatic severe PR, defined by the presence of exercise intolerance, heart failure symptoms, or arrhythmias attributable to RV dysfunction.
- Asymptomatic severe pulmonary regurgitation, in the presence of one or more of the following criteria:
  - Progressive RV dilatation (e.g., RVEDVi > 150–170 mL/m^2^ and/or RVESVi > 80–90 mL/m^2^;
  - Progressive decline in RV or LV systolic function;
  - Worsening tricuspid regurgitation;
  - RV outflow tract obstruction with RV systolic pressure exceeding two-thirds of systemic pressure;
  - Sustained atrial or ventricular arrhythmias;
  - Progressive QRS prolongation, particularly approaching or exceeding 180 ms.

These recommendations are largely based on studies that established volumetric thresholds beyond which the likelihood of normalization of RV size after PVR decreases [21,24].

### 5.2. Early Versus Deferred PVR: What Do the Data Actually Show?

Despite widespread use of volumetric cut-offs, there is no randomized comparison of “early” vs. “late” PVR. Most data come from retrospective cohorts with substantial confounding by indication [11,14].

Some studies suggest that earlier PVR (at smaller RV volumes, younger age, or shorter PR duration) yields more complete reverse remodelling and perhaps better exercise capacity [21,28,29]. Others, however, have failed to demonstrate clear differences in hard outcomes and have even suggested that the greatest long-term benefit (in terms of freedom from death and sustained VT) may occur in patients with more advanced disease at the time of PVR who have more to gain [24].

Moreover, observational work indicates that RV volumes in many asymptomatic patients with “severe” dilatation (e.g., RVEDVi 150–170 mL/m^2^) progress very slowly over time, sometimes within the range of CMR measurement variability, and that exercise capacity may remain stable for years [14]. This challenges the notion that a fixed volumetric threshold alone should trigger intervention in all ToF patients.

### 5.3. Arrhythmic Outcomes and Sudden Death

Whether PVR reduces the risk of malignant ventricular arrhythmias remains controversial. Some studies have reported reductions in QRS duration and ventricular ectopy after PVR, and early work suggested that surgery might decrease arrhythmia propensity [4,5]. However, larger cohorts and meta-analyses have not demonstrated a consistent reduction in sustained VT or sudden death attributable solely to PVR [5,8,24].

The INDICATOR cohort and subsequent analyses have highlighted that risk is more strongly driven by RV hypertrophy (mass-to-volume ratio), biventricular dysfunction, atrial arrhythmias, QRS duration, and advanced age rather than by RV volume per se [17,24]. In this context, PVR can be seen as one tool to reduce RV volume and possibly QRS duration but is not a stand-alone cure for the arrhythmogenic substrate—particularly given the irreversibility of surgical scarring and fibrosis.

### 5.4. Contemporary Perspectives and Emerging Scepticism

Recent narrative and systematic reviews have increasingly questioned the traditional reliance on simple right ventricular (RV) volume thresholds for guiding pulmonary valve replacement (PVR) [14,24,29].

Leonardi and colleagues, for example, note that only a minority of patients with repaired tetralogy of Fallot (TOF) demonstrate genuinely progressive RV dilatation and dysfunction, while many individuals with RVEDVi values around 150–160 mL/m^2^ and preserved systolic function remain clinically stable and physically active for years. They caution that the over-emphasis on volumetric cut-offs may lead to unnecessary intervention in asymptomatic patients, exposing them to prosthetic valve failure and repeat procedures, whereas a distinct subgroup exhibits early adverse myocardial remodelling—such as elevated extracellular volume, reduced RV mass-to-volume ratio, or diastolic abnormalities—despite relatively modest RV volumes, and may warrant earlier consideration for PVR than volume criteria alone would imply.

In parallel, Pula and Harris characterize the search for the “optimal timing” of PVR in TOF as a “holy grail,” emphasizing that, although observational datasets are extensive, high-level evidence validating specific thresholds is still lacking and that future efforts should shift from single-parameter triggers toward more integrated, multimodal risk-stratification models [11].

## 6. Modifiers of Remodelling and Timing Decisions

### 6.1. Age at PVR

Younger age at PVR is generally associated with a greater potential for reverse remodelling, particularly in children and adolescents [21]. On the other hand, very early PVR in childhood may result in multiple reinterventions over a lifetime. Some studies suggest that RV and LV mechanics respond less favourably when PVR is performed in older adults who may have more advanced fibrosis and RV hypertrophy [22,24].

Age therefore acts as a two-edged sword: delayed PVR in an older adult may yield limited functional recovery, yet frequent, early PVR in younger patients raises cumulative prosthesis-related risks. Longitudinal data stratified by age at PVR are crucial for balancing these competing concerns.

### 6.2. RV Hypertrophy, Fibrosis, and Diastolic Function

The RV mass-to-volume ratio ≥ 0.3 g/mL has emerged as a powerful predictor of death and VT in repaired TOF [16]. This suggests that hypertrophic remodelling under pressure and volume load represents a critical step towards adverse outcomes. Similarly, severe RV diastolic dysfunction and elevated RV end-diastolic pressure have been associated with heart failure and transplant-free survival [19].

Diffuse myocardial fibrosis, reflected by increased ECV, appears to track with PR severity, RV dilatation, and arrhythmia and may improve (but not normalize) after PVR [13,19]. Histological studies have shown that greater fibrosis and older age at PVR predict worse post-operative remodelling and outcomes [13]. These observations imply that timing strategies should consider not just RV size but also markers of myocardial health. These limitations are particularly relevant for the RV, where current T1 mapping and extracellular volume (ECV) techniques are less robust than for the left ventricle (LV). Therefore, RV ECV values should be interpreted with caution alongside other structural, functional, and clinical parameters rather than be used in isolation to inform decisions about the timing of PVR.

### 6.3. LV Involvement and Biventricular Mechanics

LV dysfunction and abnormal LV strain are consistently linked to adverse outcomes in repaired TOF [3,17]. Some patients demonstrate disproportionately impaired LV mechanics despite modest RV dilatation, perhaps due to septal scarring, myocardial fibrosis, or long-standing dyssynchrony. In such cases, earlier intervention to reduce RV volume may help preserve LV function, even if conventional RV thresholds are not yet met. On the other hand, when LV function is already severely compromised, PVR alone may be insufficient to alter prognosis.

### 6.4. Electrical Markers

Clues to arrhythmogenic substrates can be provided by QRS duration, QRS fragmentation, QT/QT dispersion, and advanced electroanatomical mapping [4,18]. While PVR may reduce the QRS duration modestly, particularly when large RVOT aneurysms are resected, this effect is inconsistent and may not translate into reduced arrhythmic events [8,24]. Prolonged QRS duration (>180 ms) remains an important risk marker, but its utility for timing PVR is limited unless interpreted alongside structural and functional data.

## 7. Discussion

### 7.1. Why Is Optimal Timing Still Unresolved?

Despite the rich literature, several factors explain why the “optimal timing” of PVR remains contentious. First, the absence of randomized trials or rigorously matched comparisons means that nearly all available evidence is derived from observational cohorts in which timing was determined by clinician judgement, local practice patterns, and evolving guidelines; as a result, substantial confounding by indication makes it difficult to determine whether observed outcomes reflect the timing of the intervention, baseline patient risk, or unmeasured variables. Further complicating the interpretation is the heterogeneity of surgical techniques and prosthetic valves across the decades spanned by published studies, during which, approaches to TOF repair, peri-operative care, valve technology, and follow-up protocols evolved considerably. As a result, cross-era comparisons are inherently imprecise. Imaging advances contribute another layer of complexity: early volumetric thresholds were established at a time when CMR acquisition, analysis, and reference standards were far less standardized than today, meaning that improvements in image resolution and post-processing may alter how historical cut-offs should be interpreted. Additionally, PVR seeks to influence multiple clinically relevant endpoints, including symptoms, exercise capacity, quality of life, ventricular remodelling, arrhythmias, heart failure, and survival, and an intervention that benefits one domain may have neutral or uncertain effects on another. Finally, biological variability and divergent disease trajectories among patients with severe PR challenge any attempt to apply uniform metrics; some individuals manifest early fibrosis or limited contractile reserve, whereas others tolerate substantial RV dilatation with preserved function for decades; refs. [15,24] underscore why a single volumetric threshold is unlikely to suit all patients.

### 7.2. Towards a Multiparametric, Individualized Model (Figure 1)

The emerging consensus is that PVR timing should be guided by an integrated assessment rather than reliance on any single metric. A practical yet more nuanced framework would consider symptoms and functional capacity, emphasizing objective exercise testing such as peak VO_2_ and ventilatory efficiency rather than self-reported activity alone.

**Figure 1:**
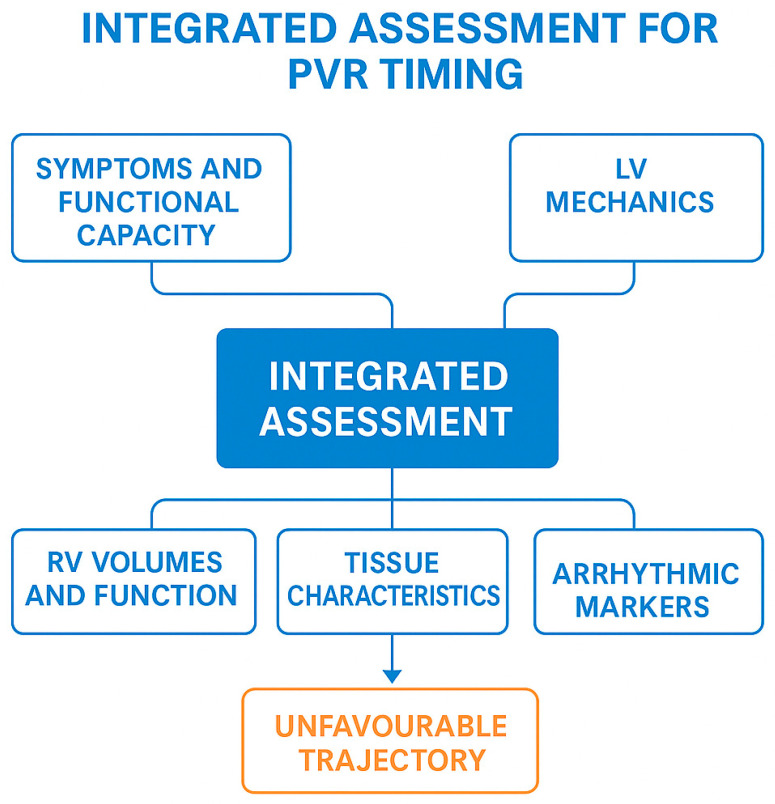
Integrated assessment for PVR timing.

It would incorporate RV volumes and function, including RVEDVi, RVESVi, RVEF, RV mass index, and the RV mass-to-volume ratio, alongside LV mechanics such as LVEF, LV strain, and diastolic indices, which are particularly relevant in the presence of septal scarring or dyssynchrony. Tissue characteristics—most notably focal late gadolinium enhancement, especially when assessed with 3D techniques in the RVOT and septal regions, and extracellular volume, where available—would help identify diffuse or early fibrosis.

Arrhythmic markers, including QRS duration and morphology, ambulatory or device-recorded arrhythmias, and electroanatomic mapping in selected patients, add further prognostic resolution, as do haemodynamic factors such as RV systolic and diastolic pressures, restrictive physiology, RVOT gradient, and associated valvular lesions.

Cardiopulmonary exercise testing (CPET) and other objective exercise assessments provide valuable additional information when monitoring patients with pulmonary valve disease. Parameters such as peak oxygen uptake (VO_2_ peak), ventilatory efficiency (VE/VCO_2_ slope), and exercise-induced arrhythmias reflect the integrated response of the cardiovascular, pulmonary, and peripheral systems and can reveal functional limitations that are not evident from resting imaging parameters alone [5,8]. Several studies have demonstrated only modest correlations between RV volumes and exercise capacity, highlighting the importance of CPET in improving risk stratification and determining the timing of PVR, particularly in asymptomatic or mildly symptomatic patients [15,24]. Accordingly, exercise testing should be considered as part of a comprehensive, multiparametric follow-up strategy rather than as a standalone criterion for intervention.

Pulmonary valve disease in patients with repaired TOF should be considered within a distinct clinical and pathophysiological framework compared with other CHD. In TOF, chronic PR typically coexists with a complex surgical substrate, including right ventricular outflow tract scarring, patch material, and conduction abnormalities, which contribute to adverse electrical and mechanical remodelling and increased arrhythmic risk [4,17,18]. In contrast, pulmonary valve dysfunction in other congenital conditions, such as isolated pulmonary stenosis or conduit degeneration following biventricular repair, may involve different patterns of ventricular loading and remodelling. Therefore, thresholds and timing strategies derived from TOF populations should not be extrapolated indiscriminately to other congenital heart diseases (CHDs), emphasizing the need for diagnosis-specific and anatomy-informed evaluation.

Beyond isolated cut-offs, the timing of PVR is best determined by integrating multiple modifying factors derived from the evidence discussed in Section 4 and Section 5. These factors include the RV remodelling trajectory rather than absolute volumes alone, biventricular dysfunction, progressive QRS prolongation or arrhythmias, declining exercise capacity, and, where available, the extent of myocardial fibrosis. It is important to note that these parameters do not carry equal weight for all patients. For instance, a patient with modest RV dilatation accompanied by rapid volumetric progression or deteriorating functional capacity may require earlier intervention than an individual with larger but stable RV volumes who is otherwise asymptomatic. This comparative, judgement-based framework highlights the need to move away from rigid thresholds towards a more personalized, multiparametric approach.

In practical terms, the following modifiers may shift the balance towards earlier intervention (Table 1): (i) rapid progression of right ventricular (RV) volumes on serial imaging; (ii) declining RV ejection fraction (RVEF) or emerging left ventricular (LV) dysfunction; (iii) increasing QRS duration or arrhythmia burden; (iv) worsening exercise capacity on cardiopulmonary exercise testing (CPET); and (v) imaging evidence of diffuse myocardial fibrosis.

Conversely, factors favouring a more conservative strategy include stable RV size and function; preserved exercise tolerance; an absence of arrhythmias; and patient-specific considerations regarding lifetime procedural burden.

### 7.3. Balancing the Burden of Repeated Interventions

Any timing strategy must consider the finite durability of biological pulmonary prostheses (surgical or transcatheter), particularly in young, physically active patients. Each repeat PVR carries cumulative risks: endocarditis, conduit/valve degeneration, stent fracture, vascular complications, and, eventually, limited surgical options after multiple sternotomies [5,8,26].

This suggests that an aggressive, prophylactic “replace early and often” strategy is not appropriate for all asymptomatic patients. Instead, timing should aim to maximize the net benefit window, intervening before irreversible damage but avoiding unnecessary early prosthesis implantation in those whose remodelling trajectory is benign. Achieving this balance is the central challenge for future research.

To summarize the practical trade-offs discussed above, Table 2 compares potential advantages and disadvantages of “early” versus “deferred” pulmonary valve replacement based on the available evidence.

Importantly, “early” and “deferred” strategies represent ends of a continuum rather than binary choices. In practice, timing is guided by serial trends and the integration of multiple parameters, including RV volumes and function, LV involvement, QRS duration and arrhythmia burden, exercise capacity (e.g., CPET), and myocardial tissue markers, where available. Given the absence of randomized trials and the heterogeneity of repaired TOF/RVOT phenotypes, the most defensible approach is an individualized decision that balances the risk of irreversible myocardial remodelling against the cumulative lifetime burden of valve reintervention.

## 8. Conclusions

PVR is an effective palliative intervention for chronic PR after TOF and other RVOT repairs. It reliably abolishes PR, reduces RV volume, improves symptoms, and often improves LV function. Nonetheless, it does not fully restore normal anatomy or eliminate arrhythmia risk, and its benefits are time-limited by prosthetic valve degeneration and recurrent RV loading.

Current evidence does not support a single universal volumetric or ECG threshold for “optimal” PVR timing. Instead, risk appears to be shaped by a complex interplay of RV size, RV hypertrophy, biventricular systolic and diastolic function, myocardial fibrosis, electrical remodelling, and age. In light of this, timing decisions should be individualized, based on comprehensive multiparametric evaluation and shared decision-making with patients, ideally in experienced adult congenital heart disease centres.

Future work must move beyond simple volumetric cut-offs to integrate advanced imaging, electrophysiology, and possibly machine-learning models into clinical algorithms, with the long-term goal of identifying those patients for whom PVR meaningfully alters the natural history of disease.

## 9. Future Directions and Research Recommendations

Based on the current literature and its limitations, the following priorities for future research can be proposed in Table 3.

In summary, the field is transitioning from a relatively crude, volume-driven approach towards a more accurate, precision-medicine model of PVR timing. Realizing this vision will require coordinated multicentre efforts, robust phenotyping, and a willingness to challenge long-standing dogmas about “severe” RV dilatation and “late” intervention.

## Figures and Tables

**Table 1:** Clinically decisive elements for evaluating indications for PVR and the key items to include in clinical and imaging reports for repaired TOF and right ventricular outflow tract (RVOT) lesions.

Domain	Clinically Decisive Elements	Key Items to Include in Imaging Reports
Clinical status	Symptoms (NYHA class, exercise intolerance), heart failure signs, and syncope; changes over time.	Current symptoms and trajectory; medications; prior interventions; and planned concomitant procedures.
ECG/rhythm	QRS duration and trend; atrial arrhythmias; and ventricular ectopy or ventricular tachycardia.	QRS duration (ms), with prior comparison; rhythm documentation; and Holter summary, if available.
Exercise testing	Objective functional assessment; exercise-induced arrhythmias.	Peak VO_2_, VE/VCO_2_ slope, O_2_ pulse, and chronotropic response; exercise-induced arrhythmias-
Pulmonary regurgitation	Severity of PVR and RV volume loading.	PR fraction by CMR; RVOT gradient; and surrogate echocardiographic parameters if needed.
RV size and function (CMR)	RV dilatation and systolic function; progression over time.	RVEDVi, RVESVi, RVEF, and RV mass index or mass-to-volume ratio; comparison with prior studies.
LV size and function	LV dysfunction or adverse mechanics.	LVEF, LV volumes; septal motion; and LV strain, if available.
Associated lesions	Tricuspid regurgitation severity; RVOT obstruction; pulmonary artery branch stenosis; and residual shunts.	Quantitative tricuspid regurgitation; RV systolic pressure estimate; and pulmonary artery flow distribution.
Tissue characterization (CMR)	Myocardial scar burden and fibrosis as modifiers of risk and remodelling.	Presence and extent of LGE (location); T1 mapping/ECV values with limitations for RV explicitly stated.
Timing considerations	Integrated assessment rather than reliance on single cut-offs.	Imaging-based decision support summary; anatomical suitability for transcatheter versus surgical PVR.

Abbreviations: CMR, cardiovascular magnetic resonance; ECV, extracellular volume; LGE, late gadolinium enhancement; LV, left ventricle; NYHA, New York Heart Association; PR, pulmonary regurgitation; PVR, pulmonary valve replacement; QRS, electrocardiographic Q, R and S wave; RV, right ventricle; RVEDVi, right ventricular end-diastolic volume index; RVESVi, right ventricular end-systolic volume index; RVOT, right ventricular outflow tract.

**Table 2:** Early versus deferred PVR: potential advantages, drawbacks, and evidence considerations.

Strategy	Potential Advantages	Potential Drawbacks/Risks	Evidence Context/Interpretation
Early PVR (before advanced RV remodelling)	Greater likelihood of RV reverse remodelling and normalization of RV volumes; potential to preserve biventricular function; may reduce progressive QRS widening and electrical remodelling; and may improve symptoms/exercise tolerance in selected patients	Higher lifetime burden of reinterventions due to limited prosthesis durability; cumulative procedural risk; may expose some patients to intervention before irreversible changes would have occurred; and not clearly proven to reduce long-term arrhythmic events or mortality	Evidence largely observational; thresholds derive mainly from CMR volumetric cut-offs predicting incomplete reverse remodelling rather than hard endpoints; and benefit greatest in patients with progressive remodelling or additional risk markers
Deferred PVR (watchful waiting)	Avoids premature implantation; may reduce total lifetime number of valve interventions; allows for later selection of most appropriate device; and appropriate in stable asymptomatic patients	Risk of crossing threshold beyond which RV reverse remodelling is incomplete; progression of fibrosis, hypertrophy, and diastolic dysfunction; progressive QRS prolongation and arrhythmia substrate; and intervention at older age with more comorbidity	Requires close surveillance and multiparametric triggers; delaying too long may reduce likelihood of normalization after PVR; no universal cut-off; and decisions remain individualized

Abbreviations: CMR, cardiovascular magnetic resonance; PVR, pulmonary valve replacement; and RV, right ventricle.

**Table 3:** Future research directions and recommendations.

Priority Area	Key Research Directions
Prospective, multicentre timing studies	Establish carefully controlled prospective cohorts comparing “early” versus “deferred” PVR using standardized inclusion criteria and imaging protocols. Use propensity-matched, quasi-experimental, or instrumented designs to reduce confounding when randomization is not feasible.
Integration of advanced imaging biomarkers	Incorporate 3D LGE, T1 mapping/ECV, and strain imaging into large longitudinal registries. Define robust, reproducible thresholds for these biomarkers that predict adverse outcomes or remodelling failure and determine whether PVR modifies their trajectories.
Refined risk stratification models	Develop and validate multiparametric risk scores combining clinical, imaging, and electrophysiological variables (e.g., RV mass-to-volume ratio, LV strain, ECV, QRS duration, and arrhythmia burden). Explore machine-learning approaches to capture nonlinear interactions and heterogeneity in patient trajectories.
Long-term comparative effectiveness of surgical vs. transcatheter PVR	Compare long-term remodelling, durability, reintervention rates, endocarditis risk, and quality of life between surgical PVR and TPVR in comparable populations. Account for anatomy and age differences that affect outcomes.
Focus on myocardial health rather than volume alone	Investigate temporal relationships among PR, RV dilatation, fibrosis, hypertrophy, and diastolic dysfunction. Determine whether early PVR reduces diffuse fibrosis progression and whether this improves survival or arrhythmia outcomes.
Age- and phenotype-specific strategies	Stratify recommendations by age group (children, adolescents, young adults, and older adults) and by anatomical subtype (e.g., transannular patch vs. conduit, native RVOT vs. prior PVR). Evaluate whether certain phenotypes (e.g., high RV mass-to-volume ratio, elevated ECV) benefit from earlier PVR irrespective of RV volumes.
Patient-reported outcomes and shared decision-making	Integrate quality of life, physical activity, and shared decision-making patient preferences into outcome assessment. Study how patients value trade-offs between procedural risks, prosthesis durability, exercise capacity, and arrhythmia risk to inform shared decision-making frameworks.
Global collaboration and data sharing	Expand international registries (e.g., INDICATOR and related initiatives) with standardized data collection and shared, de-identified datasets. Build sufficiently large datasets to enable robust, adequately powered analyses of timing-related outcomes.

## Data Availability

No new data were created or analyzed in this study.

## Supplementary Materials

The following supporting information can be downloaded at: https://www.mdpi.com/article/10.3390/jcm15031295/s1, Table S1. Landmark studies and meta-analyses on ventricular remodelling after PVR [30]; Table S2. Surgical PVR cohorts evaluating ventricular remodelling; Table S3. Transcatheter PVR cohorts [31].

## References

[1] Geva T. (2006). Indications and timing of pulmonary valve replacement after tetralogy of Fallot repair. Semin. Thorac. Cardiovasc. Surg. Pediatr. Card. Surg. Annu..

[2] Geva T. (2011). Repaired tetralogy of Fallot: The roles of cardiovascular magnetic resonance in evaluating pathophysiology and for pulmonary valve replacement decision support. J. Cardiovasc. Magn. Reson..

[3] Cheung E.W., Liang X.C., Lam W.W., Cheung Y.F. (2009). Impact of right ventricular dilation on left ventricular myocardial deformation in patients after surgical repair of tetralogy of Fallot. Am. J. Cardiol..

[4] A Gatzoulis M., Balaji S., A Webber S., Siu S.C., Hokanson J.S., Poile C., Rosenthal M., Nakazawa M., Moller J.H., Gillette P.C. (2000). Risk factors for arrhythmia and sudden cardiac death late after repair of tetralogy of Fallot: A multicentre study. Lancet.

[5] Ferraz Cavalcanti P.E., Sá M.P.B.O., Santos C.A., Esmeraldo I.M., de Escobar R.R., de Menezes A.M., Azevedo O.M., Vasconcelos Silva F.P., Lins R.F., Lima R.D. (2013). Pulmonary valve replacement after operative repair of tetralogy of Fallot: A meta-analysis and meta-regression of 3,118 patients from 48 studies. J. Am. Coll. Cardiol..

[6] Harrild D.M., Marcus E., Hasan B., Alexander M.E., Powell A.J., Geva T. (2013). Impact of transcatheter pulmonary valve replacement on biventricular strain and synchrony assessed by cardiac magnetic resonance feature tracking. Circ. Cardiovasc. Interv..

[7] Van den Eynde J., Sá M.P.B.O., Vervoort D., Roever L., Meyns B., Budts W. (2022). Pulmonary valve replacement in tetralogy of Fallot: An updated meta-analysis. Ann. Thorac. Surg..

[8] Baumgartner H., Bonhoeffer P., De Groot N.M., de Haan F., Deanfield J.E., Galie N., Gatzoulis M.A., Gohlke-Baerwolf C., Kaemmerer H. (2010). ESC guidelines for the management of grown-up congenital heart disease. Eur. Heart J..

[9] Pula G., Harris K.C. (2024). Optimal timing of pulmonary valve replacement—The holy grail in tetralogy of Fallot. Can. J. Cardiol..

[10] Chen C.A., Dusenbery S.M., Valente A.M., Powell A.J., Geva T. (2016). Myocardial extracellular volume fraction adds prognostic information in adults with repaired tetralogy of Fallot. J. Cardiovasc. Magn. Reson..

[11] Leonardi B., Stambach D., Büchel E.V. (2024). Repaired tetralogy of Fallot: Have we understood the right timing of pulmonary valve replacement?. J. Clin. Med..

[12] Valente A.M., Geva T. (2017). How to image repaired tetralogy of Fallot. Circ. Cardiovasc. Imaging.

[13] Heng E.L., Gatzoulis M.A., Uebing A., Sethia B., Uemura H., Smith G.C., Diller G.-P., McCarthy K.P., Ho S.Y., Li W. (2017). Immediate and mid-term cardiac remodeling after surgical pulmonary valve replacement in adults with repaired tetralogy of Fallot. Circulation.

[14] Valente A.M., Gauvreau K., Assenza G.E., Babu-Narayan S.V., Schreier J., A Gatzoulis M., Groenink M., Inuzuka R., Kilner P.J., Koyak Z. (2014). Contemporary predictors of death and sustained ventricular tachycardia in patients with repaired tetralogy of Fallot enrolled in the INDICATOR cohort. Heart.

[15] Davlouros P.A., Kilner P.J., Hornung T.S., Li W., Francis J.M., Moon J.C., Smith G.C., Tat T., Pennell D.J., Gatzoulis M.A. (2002). Right ventricular function in adults with repaired tetralogy of Fallot assessed with cardiovascular magnetic resonance imaging. J. Am. Coll. Cardiol..

[16] Tzemos N., Harris L., Carasso S., Dos Subira L., Greutmann M., Provost Y., Redington A.N., Rakowski H., Siu S.C., Silversides C.K. (2009). Adverse left ventricular mechanics in adults with repaired tetralogy of Fallot. Am. J. Cardiol..

[17] Ghonim S., Ernst S., Keegan J., Giannakidis A., Spadotto V., Voges I., Smith G.C., Boutsikou M., Montanaro C., Wong T. (2020). Three-dimensional late gadolinium enhancement cardiovascular magnetic resonance predicts inducibility of ventricular tachycardia in adults with repaired tetralogy of Fallot. Circ. Arrhythmia Electrophysiol..

[18] Egbe A.C., Pellikka P.A., Miranda W.R., Bonnichsen C.R., Reddy Y.N.V., Borlaug B.A. (2020). Echocardiographic predictors of severe right ventricular diastolic dysfunction in tetralogy of Fallot: Relations to patient outcomes. Int. J. Cardiol..

[19] Nonaka H., Rätsep I., Obonyo N.G., Suen J.Y., Fraser J.F., Chan J. (2024). Current trends and latest developments in echocardiographic assessment of right ventricular function: Load dependency perspective. Front. Cardiovasc. Med..

[20] Burkhardt B.E.U., Forte M.N.V., Durairaj S., Rafiq I., Valverde I., Tandon A. (2017). Timely pulmonary valve replacement may allow preservation of left ventricular circumferential strain in patients with tetralogy of Fallot. Front. Pediatr..

[21] Valsangiacomo Büchel E.R., Dave H.H., Kellenberger C.J., Dodge-Khatami A., Prêtre R., Berger F. (2005). Remodelling of the right ventricle after early pulmonary valve replacement in children with repaired tetralogy of Fallot: Assessment by cardiovascular magnetic resonance. Eur. Heart J..

[22] Hallbergson A., Gauvreau K., Powell A.J., Geva T. (2015). Right ventricular remodeling after pulmonary valve replacement: Early gains, late losses. Ann. Thorac. Surg..

[23] Oosterhof T., van Straten A., Vliegen H.W., Meijboom F.J., van Dijk A.P., Spijkerboer A.M., Bouma B.J., Zwinderman A.H., Hazekamp M.G., de Roos A. (2007). Preoperative thresholds for pulmonary valve replacement in patients with corrected tetralogy of Fallot using cardiovascular magnetic resonance. Circulation.

[24] Bokma J.P., Winter M.M., Oosterhof T., Mulder B.J.M., Bouma B.J. (2018). Preoperative predictors of death and sustained ventricular tachycardia after pulmonary valve replacement in repaired tetralogy of Fallot. Circulation.

[25] Discigil B., Dearani J.A., Puga F.J., Schaff H.V., Hagler D.J., Warnes C.A., Danielson G.K. (2001). Late pulmonary valve replacement after repair of tetralogy of Fallot. J. Thorac. Cardiovasc. Surg..

[26] Dobbels B., Herregods M.-C., Troost E., Van De Bruaene A., Rega F., Budts W., De Meester P. (2017). Early versus late pulmonary valve replacement in patients with transannular patch-repaired tetralogy of Fallot. Interact. Cardiovasc. Thorac. Surg..

[27] Jussli-Melchers J., Hansen J.H., Scheewe J., Attmann T., Eide M., Logoteta J. (2023). Pulmonary valve reconstruction for acquired pulmonary regurgitation in patients with treated congenital heart disease. Interdiscip. Cardiovasc. Thorac. Surg..

[28] Bing R., Dweck M.R. (2019). Myocardial T1 mapping: Methods, applications, and limitations. Heart.

[29] Sado D.M., White S.K., Piechnik S.K., Banypersad S.M., Treibel T.A., Captur G., Fontana M., Maestrini V., Flett A.S., Robson M.D. (2012). Identification and assessment of diffuse myocardial fibrosis in cardiomyopathies using T1 mapping. J. Am. Coll. Cardiol..

[30] Therrien J., Provost Y., Merchant N., Williams W., Colman J., Webb G. (2005). Optimal timing for pulmonary valve replacement in adults after tetralogy of Fallot repair. Am. J. Cardiol..

[31] McElhinney D.B., Hellenbrand W.E., Zahn E.M., McEnaney S., Cheatham J.P., Jones T.K., Lock J.E., Vincent J.A. (2010). Short- and medium-term outcomes after transcatheter pulmonary valve replacement in the US Melody valve investigational trial. Circulation.

