# Supplementary material for: Pulmonary Valve Replacement: Update on Timing and Ventricular Remodelling
Source: J Clin Med. 2026 Feb 6;15(3):1295. doi: 10.3390/jcm15031295 (PMC12898050; doi:10.3390/jcm15031295)
Supplement: Supplementary file 1 [file jcm-15-01295-s001.zip › jcm-4099005-supplementary.pdf]

Supplementary Materials:

Table S1. Landmark studies and meta-analyses on ventricular remodelling after PVR

Study	Design / Population	N	Region	Intervention	Imaging	Follow-up	Aim	Main remodelling findings	Notes / correlations
Büchel et al. [21].	Paediatric repaired TOF	30	Europe	Surgical PVR	CMR	~6 months	Early reverse remodelling	RVEDVi and RVESVi reduced ~30–40%; modest RVEF improvement	Early PVR associated with greater reverse remodelling
Hallbergson et al. [22].	Repaired TOF	101	NR	Surgical PVR	CMR	Up to 10 years	Time course of reverse remodelling	PR fraction ~49%→~3%; RVEDVi ↓~39%; RVESVi ↓~33%; volumes stable ~5–6 years	Later re-enlargement beyond ~7 years
Oosterhof et al. [23].	Adults with repaired TOF	71	N	Surgical PVR	CMR	NR	Point of no return	Pre-op RVEDVi <160 mL/m² or RVESVi <82 mL/m² more likely to normalise volumes	Pre-op volumes predict likelihood of normalisation
Ferraz Cavalcanti et al. [5].	Meta-analysis	3118	Mixed	Surgical/overall	Mixed	Early outcomes	Aggregate remodelling effect	Improved RV volumes, RVEF, LVEF, QRS, NYHA; low early mortality	Confirms consistent reverse remodelling
Van den Eynde et al. [7].	Updated meta-analysis	6900	Mixed	Overall	Mixed	NR	Updated aggregate effects	Consistent reverse remodelling; heterogeneity in long-term endpoints	Highlights evidence gaps
Therrien et al. [30].	Adults with repaired TOF	41 suitable here?	North America	Surgical PVR	CMR	~1 year	Effect of PVR on RV remodelling	Significant reduction in RV volumes; modest functional improvement	Adult remodelling less complete than in paediatric cohorts

Abbreviations: CMR, cardiovascular magnetic resonance; Echo, echocardiography; LV, left ventricle; LVEF, left ventricular ejection fraction; N, number of patients; NR, not reported; PR, pulmonary regurgitation; PVR, pulmonary valve replacement; RV, right ventricle; RVEDVi, right ventricular end-diastolic volume index; RVESVi, right ventricular end-systolic volume index; RVEF, right ventricular ejection fraction; RVOT, right ventricular outflow tract; TOF, tetralogy of Fallot.

Table S2. Surgical PVR cohorts evaluating ventricular remodelling

Study	Design	Diagnosis	N	Age	Region	Valve type	Imaging	Follow-up	Main outcomes	Key correlations
Büchel et al. [21].	Prospective/retrospective	TOF	NR	~14 years	Europe	NR	CMR	6 months	RV volumes ↓ 30–40%	Timing influences remodelling
Hallbergson et al. [22].	Retrospective	TOF	101	NR	NR	NR	CMR	Up to 10 years	Early remodelling; later re-dilatation	Durability linked to recurrent PR
Heng et al. [13].	Adults	TOF	62	NR	NR	NR	CMR	NR	Immediate/mid-term remodelling	Adults less complete response
Discigil et al. [25].	Late PVR	TOF	56	NR	NR	NR	Mixed	NR	Symptomatic/volumetric improvement	Earlier better remodelling
Dobbels et al. [26].	Early vs late	TOF	51	NR	NR	NR	Mixed	NR	Early vs late comparison	Timing impacts trajectory

Abbreviations: CMR, cardiovascular magnetic resonance; Echo, echocardiography; N, number of patients; NR, not reported; PR, pulmonary regurgitation; PVR, pulmonary valve replacement; RV, right ventricle; RVOT, right ventricular outflow tract; TAP, transannular patch; TOF, tetralogy of Fallot.

Table S3. Transcatheter PVR cohorts

Study	Design	Population	N	Region	Device	Imaging	Follow-up	Key findings	Notes
Harrild et al. [6].	TPVR cohort	Repaired TOF / RVOT conduit	26	NR	TPVR	CMR feature tracking	NR	Improved biventricular strain	Mechanics improve
McElhinney et al. [31].	Multicentre registry	RVOT conduit dysfunction	136	North America/Europe	Melody valve	Echo/CMR	~1 year	Significant reduction in PR and RV volumes	Large real-world TPVR evidence

Abbreviations: CMR, cardiovascular magnetic resonance; Echo, echocardiography; N, number of patients; NR, not reported; PR, pulmonary regurgitation; PVR, pulmonary valve replacement; RV, right ventricle; RVOT, right ventricular outflow tract; TPVR, transcatheter pulmonary valve replacement; TOF, tetralogy of Fallot.
